# Clinical implication of Keap1 and phosphorylated Nrf2 expression in hepatocellular carcinoma

**DOI:** 10.1002/cam4.788

**Published:** 2016-09-20

**Authors:** Jiang Chen, Yaojun Yu, Tong Ji, Rui Ma, Mingming Chen, Gaofeng Li, Feibo Li, Qiong Ding, Qingsong Kang, Diyu Huang, Xiao Liang, Hui Lin, Xiujun Cai

**Affiliations:** ^1^ Department of General SurgerySir Run Run Shaw Hospital of Zhejiang UniversityHangzhouZhejiangChina; ^2^ Life Sciences Institute and Innovation Center for Cell Signaling NetworkZhejiang UniversityHangzhouZhejiangChina; ^3^ Department of SurgeryZhejiang University Hospital, Zhejiang UniversityHangzhouZhejiangChina; ^4^ Department of General SurgeryZhejiang Putuo hospitalzhoushanZhejiangChina

**Keywords:** Hepatocellular carcinoma, Keap1, Oxidative stress, phosphorylated Nrf2 (pNrf2), prognosis

## Abstract

In this paper, variation tendency of phosphorylated Nrf2, as the activated form of native Nrf2, was studied in 107 primary hepatocellular carcinoma (HCC) specimens treated by curative hepatectomy. Moreover, the coexpression of oxidative stress markers Keap1 and pNrf2, and their association with pathological features were also evaluated based on those specimens. The results showed that preserved cytoplasmic Keap1 expression of cancer cells was observed in 59 HCCs, while reduced Keap1 expression was determined in remaining 48 ones. With regarding to nuclear pNrf2 expression, 75 HCCs were defined as high and the other 32 ones as low. There was a significant association between Keap1 and pNrf2 expression in HCCs. Higher pNrf2 expression was observed, at a more substantial proportion, in those specimens with reduced Keap1 expression, compared to those with preserved Keap1 expression. The subset with higher pNrf2 and reduced Keap1 expression was defined as pNrf2^+^ Keap1^−^. According to the analysis of prognosis, this subset was significantly associated with poor 5‐year overall survival and worse disease‐free survival in HCCs, indicating that pNrf2 and Keap1 were two‐functional biomolecules, not only the oxidative stress markers but also biomarkers for prognosis of HCCs.

## Introduction

Hepatocellular carcinoma (HCC) ranks second among the most common cancer‐related mortality in the world 1. Despite the progress of medical technology, the prognosis of HCC still remains poor and most patients experience disease recurrence and exhibit a 5‐year relative survival rate of less than 10% 2. At present, as a new molecular‐targeted therapy, sorafenib has been confirmed to be an encouraging systemic treatment against advanced HCC. However, the efficacy of sorafenib is still not satisfactory 3, 4. The appropriate use of conventional or potential treatments for hepatocellular carcinoma is still a challenge 5. For HCC, a better understanding of molecular mechanisms underlying HCC development is in urgent need.

Multiple etiological factors have been reported to be associated with the development of HCC. Among these factors, reactive oxygen species (ROS) has been a very hot topic. Relatedly, oxidative stress results from an elevation of ROS production or a decline of ROS‐scavenging capacity attracts a mass of focus from many researchers 6. In the oxidative stress pathway, there are two very important molecules, transcription factor nuclear factor erythroid 2‐related factor 2 (Nrf2) and Kelch‐like ECH‐associated protein‐1 (Keap1) 7. Upon exposure of cells to oxidative stress, Nrf2 translocates to the nucleus and is abundantly transcribed in cancer cells. There is a large body of evidence that indicated Nrf2 promoted proliferation, invasion, and chemoresistance by determining its expression in kinds of cancer including human hepatocellular carcinoma (HCC) cells 6, 8, 9. In addition, Keap1, as an oxidative stress sensor, mediates degradation of Nrf2, and the association between the aberrant Keap1 expression and the poor prognosis has been largely investigated in various malignant tumors, especially companied with dysregulation Nrf2 expression 9, 10.

Huang et al. investigated the correlation of Keap1 and Nrf2 expression and pathological features in oral squamous‐cell carcinoma (OSCC) and elucidated that the oxidative stress markers, Keap1, Nrf2, Prdx6, and CD147 were significantly correlated with each other and had prognostic value as potential therapeutic targets in OSCC 10. Luisa et al. investigated the role of Nrf2 and Keap1 in non‐small‐cell lung carcinoma (NSCLC) and found that abnormal Nrf2 and Keap1 expression was associated with worse overall survival in NSCLC patients 11. In gastric cancer, Kawasaki et al., immunohistochemically evaluated the expression of Nrf2 and assessed its clinical significance and claimed that Nrf2 expression was positively associated with aggressive tumor behavior in gastric cancer 8. In hepatocellular carcinoma, Zhang et al. reported that inhibition of Nrf2 expression inhibited proliferation by inducing apoptosis and repressed invasion, and upregulated expression of Nrf2 was correlated with tumor differentiation, metastasis, and tumor size 12. However, phosphorylated Nrf2, as an activated form of Nrf2 were rarely reported about its expression, significance, functional mechanism in HCC.

In this study, to understand the role of Keap1 and pNrf2 in HCC and to investigate the association between their expression and prognosis of HCCs, we analyzed their expression status in paratumor and tumor and elucidate their clinical implication.

## Materials and Methods

### Patient and tissue samples

A total of 107 specimens from primary HCC patients treated by curative hepatectomy at Sir Run Run Shaw hospital (Zhejiang University, Hangzhou, China), from 2008 to 2013 were used in this study. Patients were performed with irregular/segment/hemi‐ hepatectomy. Tumor tissues were dissected from macro‐ or microscopic confirmed HCC tissues, and their corresponding paratumors were isolated from liver tissues at least 2 cm away from tumors and with no microscopic tumor cells 13. The tumor specimens and paratumors were used for Western blot, qPCR and IHC analysis. 14. All resected tumors underwent detailed pathological assessment. The clinical diagnosis and evaluation of HCC were recommended by the American Joint Committee on Cancer/United International Consensus Committee (AJCC/UICC) staging system for HCC (6th edition) 15. Patient charts were carefully reviewed to obtain other clinical data such as gender, age, tumor size, tumor multiplicity, child‐pugh class, alpha‐fetoprotein (AFP), cirrhosis, virus status, Barcelona Clinic Liver Cancer (BCLC) stage, vascular invasion, metastasis, time of recurrence, and death or time of last follow‐up. Patient survival was calculated in months starting from the time of surgery. Patients with other therapeutic procedures (such as Chemotherapy, Radiotherapy, TACE, or molecular targeted therapy either preoperative or postoperative) were excluded. Written informed consent was obtained from each patient. The use of human samples was approved by the local ethical committee.

### Immunohistochemical staining

Immunohistochemical studies were performed on 107 tumor and paratumor specimens. 5‐*μ*m sections were deparaffinized in xylenes, and hydrated in alcohols. Endogenous peroxidase activity was blocked using 3% H_2_O_2_ in methanol for 10 min. The sections were then washed and antigens were retrieved using antigen‐specific methods. Then these sections were treated with 10% normal goat serum and incubated overnight at 4°C with mouse anti‐Keap1 antibody (1B4; abcam, UK; 1:150 dilution), rabbit anti‐Nrf2 antibody (abcam, UK; 1:100 dilution), or rabbit anti‐pNrf2 antibody (phospho‐ S40; abcam, UK; 1:150 dilution). The bound primary antibody was visualized with streptavidin–biotin peroxidase (Vectastain Elite ABC reagent; Vector Laboratories, CA) according to the manufacturer's instructions. Tumor specimens that were expected to have a high expression of pNrf2, which react against the specific antibodies were used as positive control tissue sections. Tumor specimens incubated with nonimmune serum were used as negative controls, to ensure that secondary antibodies did not produce background stain.

### Evaluation of IHC staining

Five fields of each specimen were selected to evaluate the results by immunohistochemical staining. Specimens were scored by two independent pathologists. Staining intensities were assessed with high‐power (×400) microscopy, and was scored as 0 (negative), 1 (weak), 2 (moderate), or 3 (strong). The proportions of tumor cells positively stained with Keap1 and pNrf2 were scored as 0 (0%), 1 (1–25%), 2 (26–50%), 3 (51–75%), or 4 (76–100%). The Q‐score was the sum of the intensity and proportion scores and ranged from 0 to 7. A Q‐score≤2 was considered negative, or reduced expression, and a Q‐score>2 was considered positive, or preserved expression. Accordingly, cytoplasmic Keap1 and nuclear pNrf2 immunostaining were assessed in 107 HCC specimens.

### Quantitative real‐time PCR (qRT‐PCR)

Total RNA was extracted from tissues using Trizol (Invitrogen, Carlsbad, CA, USA). Reverse transcription was performed according to the protocol of PrimeScript RT reagent kit (Takara, Dalian, China). Quantitative PCR was performed using SYBR premix Ex Taq (Bio‐Rad, Hercules, CA, USA). Gene expression in each sample was normalized with the house keeping gene (GAPDH) expression. Relative quantification of target gene expression was evaluated using the comparative CT method. The primers used for this study were listed in Table 1.

**Table 1:** Oligonucleotide primers

Primer	Sequence (5′ to 3′)	Location	Use
GAPDH	CGACCACTTTGTCAAGCTCA	sense	Real‐time PCR
GAPDH	TTACTCCTTGGAGGCCATGT	antisense	Real‐time PCR
Keap1	CTGGAGGATCATACCAAGCAGG	sense	Real‐time PCR
Keap1	GGATACCCTCAATGGACACCAC	antisense	Real‐time PCR
Nrf2	CATCCAGTCAGAAACCAGTGG	sense	Real‐time PCR
Nrf2	GCAGTCATCAAAGTACAAAGCAT	antisense	Real‐time PCR
NQO1	GAAGAGCACTGATCGTACTGGC	sense	Real‐time PCR
NQO1	GGATACTGAAAGTTCGCAGGG	antisense	Real‐time PCR
GCLC	GGCACAAGGACGTTCTCAAGT	sense	Real‐time PCR
GCLC	CAGACAGGACCAACCGGAC	antisense	Real‐time PCR
GCLM	CATTTACAGCCTTACTGGGAGG	sense	Real‐time PCR
GCLM	ATGCAGTCAAATCTGGTGGCA	antisense	Real‐time PCR
AKR1B10	TCAGAATGAACATGAAGTGGGG	sense	Real‐time PCR
AKR1B10	TGGGCCACAACTTGCTGAC	antisense	Real‐time PCR
AKR1C1	CTAAAAGTAAAGCTTTAGAGGCCAC	sense	Real‐time PCR
AKR1C1	ACCTGCTCCTCATTATTGTATAAATGA	antisense	Real‐time PCR

### Western blotting

Total protein was extracted using 1× RIPA buffer (sigma). The lysis buffer was supplemented with protease inhibitor cocktail (Roche). According to protein concentrations, equal amounts of protein were separated on 12% polyacrylamide gels and transferred onto 0.45 *μ*m nitrocellulose membranes (Millipore). The membranes were blocked with 5% fat‐free dry milk in TBST for 2 h, then incubated with primary antibodies, mouse anti‐Keap1 antibody (1B4; abcam, UK; 1:1000 dilution); rabbit anti‐Nrf2 antibody (abcam, UK; 1:5000 dilution); rabbit anti‐pNrf2 antibody (phospho‐ S40; abcam, UK; 1:5000 dilution) or mouse anti‐Tubulin antibody (invitrogen, USA; 1:1000 dilution). Tubulin was used as internal control. The immunoreactive blots were visualized using an enhanced chemiluminescence reagent (Clarity^™^ Western ECL Substrate, Bio‐Rad) according to the manufacturer's instructions.

### Statistical analysis

The data were analyzed by SPSS version 17.0 (SPSS Inc., Chicago, SUA). The *χ* ^2^ test or the Student's *t*‐test was used to analyze group differences. Kaplan–Meier method and the log‐rank test were used for overall survival and disease‐free survival analysis. Univariate analyses were used to examine the prognostic factors (proportional hazards regression model). A *P* < 0.05 was considered to be statistically significant.

## Results

### Clinical and pathologic variable analysis

A total of 107 patients with HCC met inclusion criteria in this cohort. The characteristics of patients and clinical characteristics of HCC included in the study were summarized in Table 2. As assessed by univariate Cox regression, no significant correlation was found between patient survival and candidate prognostic factors like age, gender, tumor size, ascites, child‐pugh class, cirrhosis, and virus status. But significant correlation was observed between patient survival and tumor multiplicity (*P* = 0.002), vascular invasion (*P* = 0.002), and metastasis (*P* = 0.005), as depicted in Table 3. Moreover, as shown in Table 4, for those HCC tissues with reduced Keap1 expression, a majority showed pNrf2 expression that was upregulated, indicating that cytoplasmic Keap1 expression significantly correlates with nuclear pNrf2 expression (*P* = 0.0103).

**Table 2:** Patient characteristics

Variable	No. of Patients (%)
No. of patients	107 (100)
Age: Median [range], y	50.15 (17–88)
Gender	
Male	86 (80.37)
Female	21 (19.63)
HBsAg status	
Negative	17 (15.88)
Positive	90 (84.12)
Child‐Pugh class	
A	101 (94.39)
B	6 (5.61)
AFP: Median [range], ng/mL	4465.2 (0–217,935)
Tumor size: Median [range], cm	4.29 (0.90–12.80)
Liver cirrhosis	
Absent	40 (37.38)
Present	67 (62.62)
ALT: Median [range], U/L	35.98 (5–245)
Tumor multiplicity	
Solitary	100 (93.46)
Multiple	7 (6.54)
Vascular invasion	
Absent	99 (92.52)
Present	8 (7.48)
Metastasis	
Absent	98 (91.59)
Present	9 (8.41)
BCLC stage	
0	15 (14.02)
A	89 (83.18)
B	2 (1.87)
C	1 (0.93)
Recurrence	
pNrf2	
high	38 (50.67)
low	10 (31.25)
Keap1	
reduced	24 (50.00)
preserved	21 (35.60)

HBsAg, hepatitis B surface antigen; AFP, alpha‐fetoprotein; ALT, alanine aminotransferase; BCLC, Barcelona Clinic Liver Cancer.

**Table 3:** Univariate analyses of factors associated with patient survival

Parameters	OS			DFS		
	HR	95% CI of HR	*P* value	HR	95% CI of HR	*P* value
Gender						
Male/female	1.257	0.398–3.971	0.696	0.628	0.268–1.475	0.286
Age (year)						
≤55/>55	1.158	0.396–3.391	0.789	0.679	0.347–1.327	0.257
HBsAg						
Positive/negative	0.899	0.248–3.258	0.871	0.852	0.336–2.164	0.737
AFP (ng/mL)						
≤20/>20	1.707	0.675–4.320	0.259	1.119	0.618–2.024	0.711
Liver cirrhosis						
Present/absent	1.780	0.671–4.723	0.247	0.653	0.352–1.212	0.177
Tumor size (cm)						
≤5/>5	0.506	0.132–1.934	0.320	0.848	0.355–2.026	0.711
Tumor multiplicity						
Solitary/multiple	0.003	0.000–0.109	0.002^a^	0.183	0.018–1.813	0.147
Vascular invasion						
Present/absent	2.123	0.000–0.016	0.002^a^	0.001	0.000–0.319	0.018^a^
Metastasis						
Present/absent	0.009	0.000–0.246	0.005^a^	0.006	0.000–0.195	0.004^a^
pNrf2						
High/Low	0.313	0.120–0.818	0.017^a^	0.445	0.241–0.821	0.010^a^
Keap1						
Reduced/Preserved	2.240	0.902–5.562	0.082	1.573	0.859–2.879	0.142

Univariate analysis, Cox proportional hazards regression model. OS, overall survival; AFP, alpha‐fetoprotein; DFS, disease‐free survival; HR, hazard ratio; CI, confidence interval.

a Significantly different.

**Table 4:** Correlation between Keap1 and pNrf2 expression in human hepatocellular carcinoma (HCC) tissues

Variable	Keap1 expression		*P* value
	Reduced	Preserved	
pNrf2 expression			
High	40	35	0.0103
Low	8	24	

### Correlation between Keap1 expression and clinical outcome

**Figure 1:**
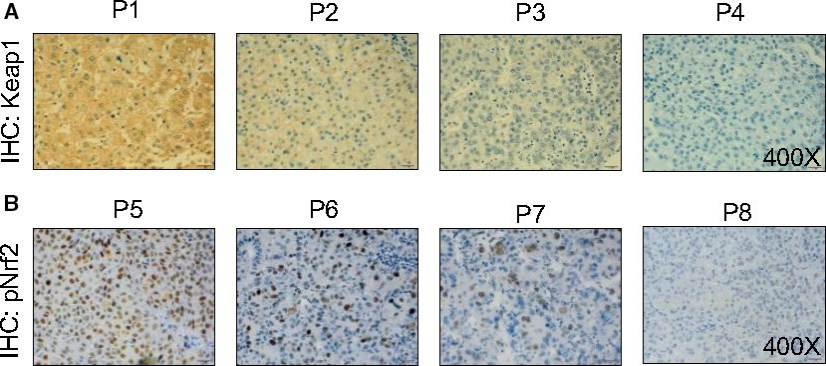
Classification of Keap1 and pNrf2 immunoreactivity in human hepatocellular carcinoma (HCC) tissue. Representative immunohistochemical staining of (P1, P5) strong, (P2, P6) moderate, (P3, P7) weak, and (P4, P8) none Keap1 (A) and pNrf2 (B) immunoreactivity in human HCC tissue.

**Figure 2:**
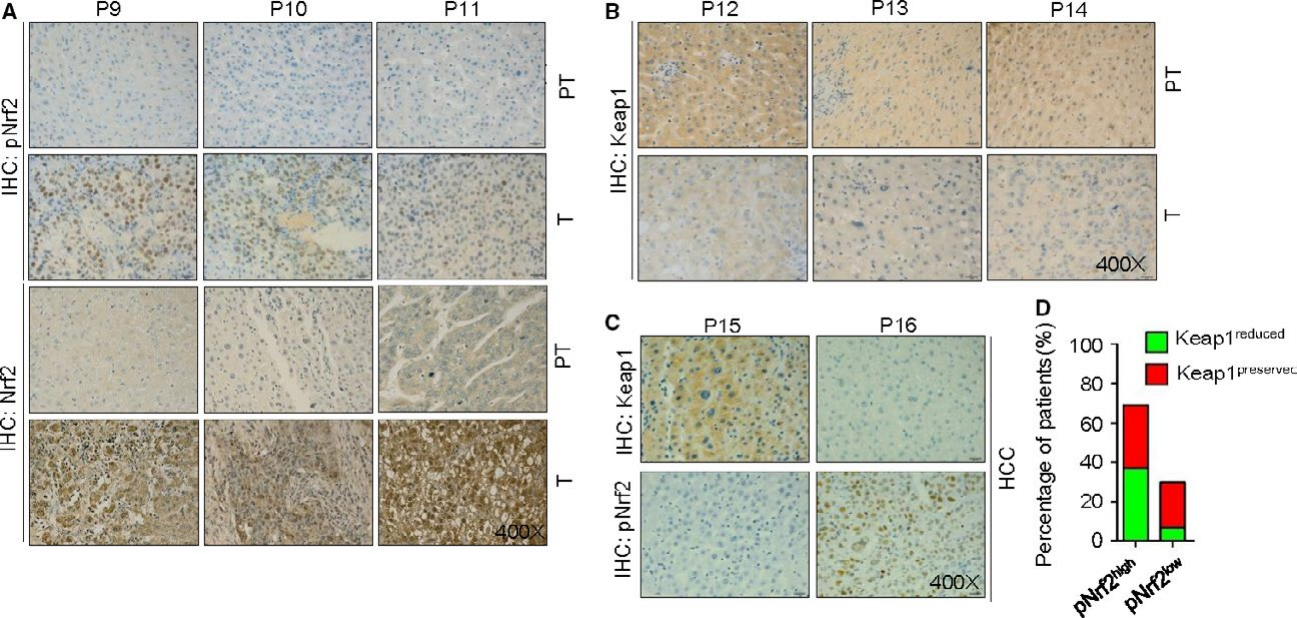
Correlation of Keap1 and pNrf2 expression in human hepatocellular carcinoma (HCC) tissues. (A) typical staining of high nuclear pNrf2 expression in tumor compare to paratumor, so does total Nrf2. (B) typical staining of reduced keap1 expression in tumor compare to paratumor. (C) Overexpression of Keap1 in HCC was correlated with low level of pNrf2, and vice versa. (D) Percentage of subtype HCC according to expression of pNrf2 and Keap1. PT: Paratumor, Adjacent noncancer.

**Figure 3:**
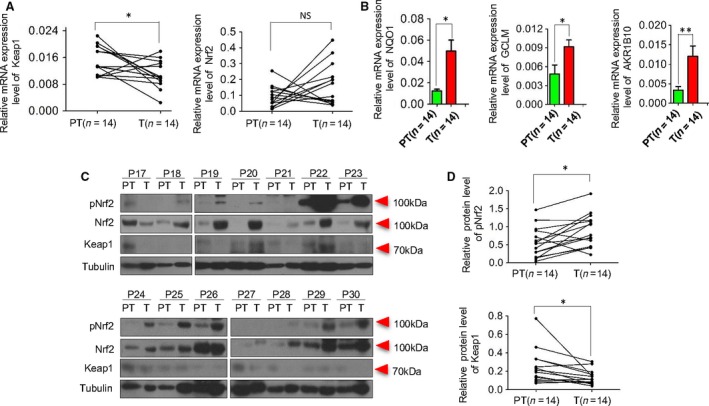
Oxidative stress markers expression in HCC patients. The mRNA levels of Keap1 and Nrf2(A), NQO1, AKR1B10 and GCLM(B) were assessed by qPCR in tumor(*n* = 14) and paratumor(*n* = 14). The expression of total protein Nrf2, pNrf2 and Keap1 were assessed by Western blot in tumor(*n* = 14) and paratumor(*n* = 14) (C). The expressions of pNrf2 and Keap1 (D) between tumor and paratumor were Compared(*n* = 14). The data were presented as *P < 0.05, **P < 0.01. PT, paratumor; T, tumor; P, patient. HCC, hepatocellular carcinoma.

**Figure 4:**
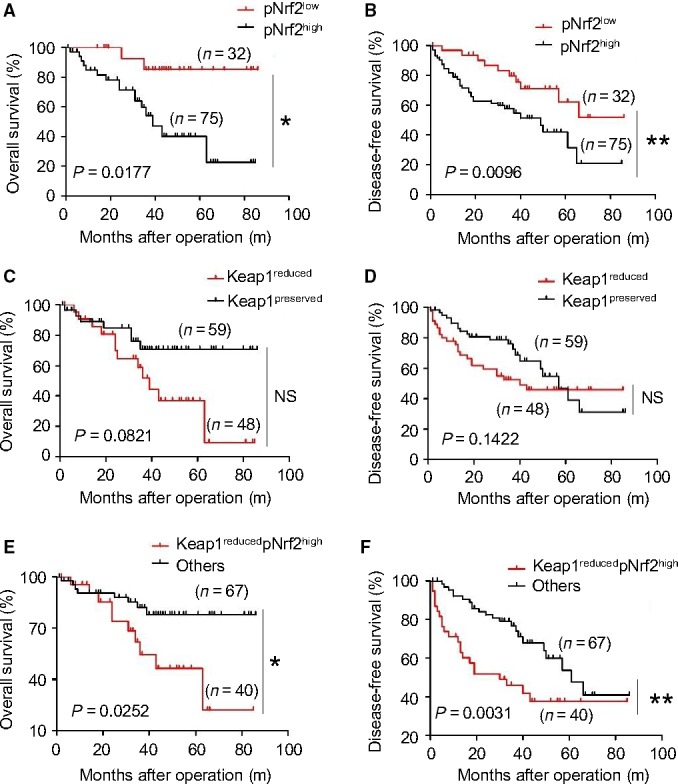
Prognostic significance of pNrf2 and Keap1 expression in 107 patients with HCC after curative resection. Cumulative overall survival (OS) time and disease‐free survival (DFS) time were calculated by Kaplan–Meier method and analyzed by log‐rank test. (A) Regarding the OS rate, significant difference was found between the group with high pNrf2 expression (*n* = 75) and the group with low pNrf2 expression (*n* = 32) (*P* = 0.0177). (B) Regarding the DFS rate, the group with high pNrf2 expression (*n* = 75) had a significantly poorer prognosis compared with the group with low pNrf2 expression (*n* = 32) (*P* = 0.0096). (C) Cumulative OS was not different between Keap1 reduced (*n* = 48) and preserved (*n* = 59) group (*P* = 0.0821). (D) Cumulative DFS was not different between Keap1 reduced (*n* = 48) and preserved (*n* = 59) group (*P* = 0.1422). (E) Cumulative OS was significant different between Keap1 reduced plus high pNrf2 expression (*n* = 40) and others (*n* = 67) group (*P* = 0.0252). (F) Cumulative DFS was significant different between Keap1 reduced plus high pNrf2 expression (*n* = 40) and preserved (*n* = 67) group (*P* = 0.0031). The data were presented as *P < 0.05, **P < 0.01.

Keap1 protein was mainly expressed in cytoplasm (Fig. 1A). As shown in Figure 2B, according to the result of IHC, reduced keap1 expression was observed in HCC tissues, while preserved keap1 expression was found in paratumors. A similar result was observed in Figure 3, according to the results of qPCR and Western blotting. In Figure 2C and D, it showed that preserved cytoplasmic Keap1 expression was always accompanied by low expression of nuclear pNrf2 (24/107), while reduced cytoplasmic Keap1 expression was always accompanied by high expression of nuclear pNrf2 (40/107) (Table 4). In addition, recurrence rate of patients with reduced Keap1 expression was higher than those with preserved Keap1. As depicted in Table 2, for 48 specimens with reduced Keap1 expression, recurrence was observed in 24 specimens, the proportion being nearly 50.0%. For 59 specimens with preserved Keap1, recurrence was observed in 21 specimens, the proportion being 35.6%. Moreover, as depicted in Table 3, reduced expression of cytoplasmic Keap1 in cancer cells was relatively associated with poor 5‐year overall survival (*P* = 0.082; hazard ratio [HR] = 2.24) and worse disease‐free survival (*P* = 0.142; HR = 1.573), and a similar result was observed in Figure 4C, D. According to the result shown in Figure 4D and F, more than 40% of Keap1^−^ patients and less than 40% of the subset of pNrf2^+^ Keap1^−^ patients lived more than 80 months after surgery. According to the analysis of prognosis, it indicated that reduced expression of cytoplasmic Keap1 was significantly associated with poor 5‐year overall survival and worse disease‐free survival in HCCs, especially with upregulated pNrf2 expression.

### Correlation between pNrf2 expression and clinical outcome

The expression of pNrf2 was primarily observed in the nucleus (Fig. 1B). The negative and positive control were shown in Figure S1D. In Figure 2A, according to the result of IHC, higher expression of pNrf2 protein was observed in HCC tissues, while reduced pNrf2 expression was found in paratumors. A similar result was observed in Figure 3C and D by Western blotting. Reported by other studies, inhibition of Nrf2 expression inhibited proliferation, and upregulated expression of Nrf2 was correlated with tumor metastasis and tumor size (12). Interestingly, as an activated form of Nrf2, the levels of pNrf2 mirror those of total Nrf2 in our study, both in tumor and paratumors (Fig. 2A and 3C). As depicted in Table 2, for 75 specimens with upregulated pNrf2 expression, recurrence was observed in 38 specimens, the proportion being nearly 50.67%. For 32 specimens with low pNrf2, recurrence was observed in 10 specimens, the proportion being 31.25%. As depicted in Figure 4A and B, univariate analysis demonstrated that upregulated expression of pNrf2 significantly correlated with poor 5‐year OS (*P* < 0.05; HR = 0.313) and DFS (*P* < 0.05; HR = 0. 4451). Furthermore, comparing the OS and DFS of pNrf2^+^ Keap1^−^ subset and other subset, it was observed that patients with high pNrf2 and reduced Keap1 expression demonstrated worse prognosis, OS (*P* < 0.05; HR = 3.133) and DFS (*P* < 0.05; HR = 2.673) (Fig. 4E and F).

### Correlation between Keap1 and nuclear pNrf2 expression

Huang et al. elucidated that the oxidative stress markers, Keap1, Nrf2, Prdx6, and CD147 were significantly correlated with each other in OSCC 10. In this study, close correlation was also found between cytoplasmic Keap1 expression and nuclear pNrf2 expression not only in tumor but also in paratumor, as shown in Figure 2 C and D and Figure S1C. In protein level, upregulated nuclear pNrf2 expression was accompanied by decreased cytoplasmic Keap1 expression. But in transcription level, no obvious changes were observed with Nrf2 (Fig. 3A). Therefore, Nrf2, in protein level, could be degraded or repressed in Keap1‐dependent manner 16. The Keap1‐Nrf2 system plays a crucial role in cellular defense against oxidative stress, which may protect not only normal cells but also cancer cells from the toxicity of reactive oxygen species 17. Various downstream genes, AKR1B10, NQO1, AKR1C1, GCLM, and GCLC were involved in this signaling pathway. In Figure 3B and Figure S1, AKR1B10, NQO1, and GCLM were activated and highly expressed as the expression of pNrf2 was upregulated, while no significant changes were observed in expression of AKR1C1 and GCLC. In addition, as oxidative stress markers, the prognostic significance of Keap1 and pNrf2 was further investigated for patients with HCC.

## Discussion

Our results evaluated the prognostic significance of expression of oxidative stress‐related genes Keap1 and pNrf2 in HCC. We found that reduced expression of cytoplasmic Keap1 with upregulated nuclear pNrf2 expression significantly correlated with poor overall survival and higher recurrence risk. It indicated that cytoplasmic Keap1 and nuclear pNrf2 could be regarded as biomarkers for prognosis of HCCs. As a crucial factor interacting with the PI3K‐AKT pathway, pNrf2 and its downstream genes promote tumor progression by regulating cell‐cycle‐related factors, and modulating mitochondrial function 18, 19, 20, 21. It confirmed that pNrf2 may be one of the underlying factors of poor prognosis of HCCs. The pNrf2 was originally regarded as a critical regulator of intracellular antioxidants through upregulating many antioxidant response element (ARE)‐containing genes. A recent study indicates that Keap1 degradation was critical for activation of the Nrf2 signaling pathway, a major defense mechanism in cancer cells 22. Hua KT and his colleagues demonstrated that Keap1 functions as a suppressor of tumor metastasis by targeting the Nrf2/S100P pathway in non‐small‐cell lung cancer cells 23. Yota Kawasaki et al. reported that Nrf2 protein was predominantly identified in the nucleus of gastric cancer cells, which was proved to be positively associated with aggressive tumor behavior 8. Joel Isohookana and his colleagues' results show that expression of Keap1, main mediating degradation of Nrf2, was a significant prognostic factor in pancreatic adenocarcinomas 24.

The action mechanism of Keap1‐Nrf2 pathway in initiation and development of HCC was still ambiguous, though a number of researchers spare no effort to conduct studies in various kinds of tumors.

Nrf2 could be degraded or repressed in two distinct manners, GSK‐3‐dependent and Keap1‐dependent. In fact, the degradation of Nrf2 in GSK‐3‐dependent manner has been gradually elucidated in recent years. Nrf2 is regulated by GSK‐3 through creation of a DSGIS‐containing phosphodegron that is recognized by beta‐TrCP 25, 26. As the PI3K‐AKT pathway inhibits GSK‐3, it is most likely it controls formation of the DSGIS phosphodegron through which beta‐TrCP enables Cul1 to ubiquitylate Nrf2. Our study has been conducted based on the Keap1‐dependent mechanism.

Some of the somatic mutations of *Keap1* gene may produce nonfunctional protein, leading to false positive immunoreactive signal for Keap1. Kornelius Schulze et al. reported that activating mutations of *NFE2L2* (encoding Nrf2) was 6% and inactivating mutations of *KEAP1* was 4% in human HCC 17. By whole‐exome and oncovirome sequencing of human HCC, a recent study demonstrated that there were frequent activating mutations of *NFE2L2* and inactivating mutations of *KEAP1* 27. These mutations lead to activation of the Keap1‐Nrf2 pathway that may protect not only normal cells but also cancer cells from the toxicity of reactive oxygen species. Moreover, researchers identified that *NFE2L2* and *KEAP1* mutations were only observed in advanced HCC suggesting that these mutations were late events in human liver carcinogenesis 17, 28, 29, 30. Nevertheless, Nam Jin Yoo et al. observed that mutated Keap1 (8.9% of HCC) was still expressed in HCC with *Keap1* mutations; among those mutated Keap1, only 25% (1/4) were false positive 31. Therefore, it indicated that only 2.225% of total HCC patients, a fairly low proportion, may produce nonfunctional protein. Similarly, Kornelius Schulze et al. demonstrated that only 4% of HCC showed *Keap1* inactivated mutation, indicating that the rate of high nonfunctional keap1 expression caused by Keap1‐inactivated mutation is even low 17. Moreover, Sean P. Cleary et al. reported that decreased expression of Keap1 was observed in 4/6 tumors with *Keap1* mutations 32. Above all, false positive immunoreactive signal for Keap1 do exist but at very low proportions.

Additionally, on the way to explore the mechanism underlying Keap1‐Nrf2 pathway, the expression of Nrf2‐target genes, as well as pNrf2 protein, play an informative role, considering their potential prognostic significance. Various downstream genes, *AKR1B10*,* NQO1*,* AKR1C1*,* GCLM*, and *GCLC* were involved in this signaling pathway. MacLeod et al. and Agyeman et al. found *aldo‐ keto reductase (AKR) 1B10* and *AKR1C1* to be major target genes of Nrf2 in human cells 33, 34. Moreover, Matkowskyj et al. have reported AKR1B10 to be upregulated in HCC 35. GCLC and GCLM were Nrf2‐target genes in humans and are linked directly to the synthesis of glutathione and thus, the oxidative stress response 36. According to the results of our study, AKR1B10, NQO1, and GCLM were activated and highly expressed as the expression of pNrf2 was upregulated, while no significant changes were observed in expression of AKR1C1and GCLC.

In this manuscript, the coexpression of oxidative stress markers Keap1 and pNrf2, and their association with pathological features were evaluated in HCC. The result showed that there was a significant association between Keap1 and pNrf2 expression in HCCs. Higher pNrf2 expression was observed, at a more substantial proportion, in those specimens with reduced Keap1 expression, compared to those with preserved Keap1 expression. According to the analysis of prognosis, the pNrf2^+^ Keap1^−^ subset was significantly associated with poor 5‐year overall survival and worse disease‐free survival in HCCs, indicating that pNrf2 and Keap1 were two‐functional biomolecules, not only the oxidative stress markers but also biomarkers for prognosis of HCCs.

According to all these recent findings, the Keap1‐Nrf2 system plays a crucial role in cellular defense against oxidative stress, which has been reported to promote cancer development and act as a biomarker to anticipate prognosis of HCCs. But little of the specific mechanisms are known about its association with carcinogenesis of HCC.

## Conclusion

In this paper, variation tendency of pNrf2, as the activated form of native Nrf2, was studied in 107 primary hepatocellular carcinoma (HCC) specimens treated by curative hepatectomy. Moreover, the coexpression of oxidative stress markers Keap1 and pNrf2, and their association with pathological features were also evaluated. The correlation between Keap1 and pNrf2 expression and clinical outcome was assessed. The result showed that there was a significant association between Keap1 and pNrf2 expression in HCCs. Higher pNrf2 expression was more likely observed in those specimens with reduced Keap1 expression. The pNrf2^+^ Keap1^−^ subset was significantly associated with poor 5‐year overall survival and worse disease‐free survival in HCCs, indicating that pNrf2 and Keap1 were two‐functional biomolecules, not only the oxidative stress markers but also biomarkers for prognosis of HCCs.

## Conflict of Interest

None declared.

## Supporting information


**Figure S1.** The mRNA levels of AKR1C1(A) and GCLC(B) were assessed by qPCR in tumor(*n* = 14) and paratumor(*n* = 14). Typical staining for high and low nuclear pNrf2 or Keap1 expression in paratumor(C). The negative and positive control for the pNrf2 (Ser40) antibody (D)

## References

[1] Jemal, A. , F. Bray , M. M. Center , J. Ferlay , E. Ward , and D. Forman . 2011 Global cancer statistics. CA Cancer J. Clin. 61:69–90. 21296855 10.3322/caac.20107

[2] Coleman, M. , D. Forman , H. Bryant , J. Butler , B. Rachet , C. Maringe , et al. 2011 Cancer survival in Australia, Canada, Denmark, Norway, Sweden, and the UK, 1995–2007 (the International Cancer Benchmarking Partnership): an analysis of population‐based cancer registry data. The Lancet 377:127–138. 10.1016/S0140-6736(10)62231-3 PMC3018568 21183212

[3] Llovet, J. M. , S. Ricci , V. Mazzaferro , P. Hilgard , E. Gane , J. F. Blanc , et al. 2008 Sorafenib in advanced hepatocellular carcinoma. New England J. Med. 359:378–390. 18650514 10.1056/NEJMoa0708857

[4] Cheng, A. L. , Y. K. Kang , Z. Chen , C. J. Tsao , S. Qin , J. S. Kim , et al. 2009 Efficacy and safety of sorafenib in patients in the Asia‐Pacific region with advanced hepatocellular carcinoma: a phase III randomised, double‐blind, placebo‐controlled trial. Lancet Oncol. 10:25–34. 19095497 10.1016/S1470-2045(08)70285-7

[5] Patel, T. , and D. Harnois . 2014 Assessment of response to therapy in hepatocellular carcinoma. Ann. Med. 46:130–137. 24716738 10.3109/07853890.2014.891355 PMC4008698

[6] Zhou, L. , Y. Yang , D. Tian , and Y. Wang . 2013 Oxidative stress‐induced 1, N6‐ethenodeoxyadenosine adduct formation contributes to hepatocarcinogenesis. Oncol. Rep. 29:875–884. 23292006 10.3892/or.2013.2227 PMC3597589

[7] Sporn, M. B. , and K. T. Liby . 2012 NRF2 and cancer: the good, the bad and the importance of context. Nat. Rev. Cancer 12:564–571. 22810811 10.1038/nrc3278 PMC3836441

[8] Kawasaki, Y. , S. Ishigami , T. Arigami , Y. Uenosono , S. Yanagita , Y. Uchikado , et al. 2015 Clinicopathological significance of nuclear factor (erythroid‐2)‐related factor 2 (Nrf2) expression in gastric cancer. BMC Cancer 15:5. 25588809 10.1186/s12885-015-1008-4 PMC4302133

[9] Jaramillo, M. C. , and D. D. Zhang . 2013 The emerging role of the Nrf2‐Keap1 signaling pathway in cancer. Genes Dev. 27:2179–2191. 24142871 10.1101/gad.225680.113 PMC3814639

[10] Huang, C. F. , L. Zhang , S. R. Ma , Z. L. Zhao , W. M. Wang , K. F. He , et al. 2013 Clinical significance of Keap1 and Nrf2 in oral squamous cell carcinoma. PLoS ONE 8:e83479. 24386210 10.1371/journal.pone.0083479 PMC3873935

[11] Solis, L. M. , C. Behrens , W. Dong , M. Suraokar , N. C. Ozburn , C. A. Moran , et al. 2010 Nrf2 and Keap1 abnormalities in non‐small cell lung carcinoma and association with clinicopathologic features. Clin. Cancer Res. 16:3743–3753. 20534738 10.1158/1078-0432.CCR-09-3352 PMC2920733

[12] Zhang, M. X. , C. Zhang , L. M. Zhang , Q. Yang , S. N. Zhou , Q. S. Wen , et al. 2015 Nrf2 is a potential prognostic marker and promotes proliferation and invasion in human hepatocellular carcinoma. BMC Cancer 15:531. 26194347 10.1186/s12885-015-1541-1 PMC4507320

[13] Yuan, Z. , Q. Zheng , J. Fan , K. X. Ai , J. Chen , and X. Y. Huang . 2010 Expression and prognostic significance of focal adhesion kinase in hepatocellular carcinoma. J. Cancer Res. Clin. Oncol. 136:1489–1496. 20151150 10.1007/s00432-010-0806-y PMC11828088

[14] Yamaoka, H. , K. Ohtsu , T. Sueda , T. Yokoyama , and E. Hiyama . 2006 Diagnostic and prognostic impact of beta‐catenin alterations in pediatric liver tumors. Oncol. Rep. 15:551–556. 16465411

[15] Lee, H. J. , J. E. Yeon , S. J. Suh , S. J. Lee , E. L. Yoon , K. Kang , et al. 2014 Clinical utility of plasma glypican‐3 and osteopontin as biomarkers of hepatocellular carcinoma. Gut. Liv. 8:177. 10.5009/gnl.2014.8.2.177 PMC3964269 24672660

[16] Niture, S. K. , R. Khatri , and A. K. Jaiswal . 2014 Regulation of Nrf2‐an update. Free Radic. Biol. Med. 66:36–44. 23434765 10.1016/j.freeradbiomed.2013.02.008 PMC3773280

[17] Schulze, K. , S. Imbeaud , E. Letouze , L. B. Alexandrov , J. Calderaro , S. Rebouissou , et al. 2015 Exome sequencing of hepatocellular carcinomas identifies new mutational signatures and potential therapeutic targets. Nat. Genet. 47:505–511. 25822088 10.1038/ng.3252 PMC4587544

[18] Taguchi, K. , I. Hirano , T. Itoh , M. Tanaka , A. Miyajima , A. Suzuki , et al. 2014 Nrf2 enhances cholangiocyte expansion in Pten‐deficient livers. Mol. Cell. Biol. 34:900–913. 24379438 10.1128/MCB.01384-13 PMC4023823

[19] Zou, Y. H. , M. Hu , J. Lee , S. M. Nambiar , V. Garcia , Q. Bao , et al. 2015 Nrf2 is essential for timely M phase entry of replicating hepatocytes during liver regeneration. Am. J. Physiol‐Gastrointest. Liver Physiol. 308:G262–G268. 25524062 10.1152/ajpgi.00332.2014 PMC4329475

[20] Itoh, K. , P. Ye , T. Matsumiya , K. Tanji , and T. Ozaki . 2015 Emerging functional cross‐talk between the Keap1‐Nrf2 system and mitochondria. J. Clin. Biochem. Nutr. 56:91–97. 25759513 10.3164/jcbn.14-134 PMC4345178

[21] Lewis, K. N. , E. Wason , Y. H. Edrey , D. M. Kristan , E. Nevo , and R. Buffenstein . 2015 Regulation of Nrf2 signaling and longevity in naturally long‐lived rodents. Proc. Natl. Acad. Sci. U S A. 112:3722–3727. 25775529 10.1073/pnas.1417566112 PMC4378420

[22] Yin, S. , and W. Cao . 2015 Toll‐Like receptor signaling induces Nrf2 pathway activation through p62‐triggered Keap1 degradation. Mol. Cell. Biol. 35:2673–2683. 26012548 10.1128/MCB.00105-15 PMC4524114

[23] Hua, K. T. , M. H. Chien , W. J. Lee , F. K. Hsieh , C. F. Li , T. Y. Cheng , et al. 2015 Keap1‐Nrf2 interaction suppresses cell motility in lung adenocarcinomas by targeting the S100P protein. Clin. Cancer Res. 21:4719–4732. 26078391 10.1158/1078-0432.CCR-14-2880

[24] Isohookana, J. , K. M. Haapasaari , Y. Soini , and P. Karihtala . 2015 Keap1 expression has independent prognostic value in pancreatic adenocarcinomas. Diagn. Pathol. 10:28. 25879528 10.1186/s13000-015-0258-4 PMC4422296

[25] Rada, P. , A. I. Rojo , S. Chowdhry , M. McMahon , J. D. Hayes , and A. Cuadrado . 2011 SCF/{beta}‐TrCP promotes glycogen synthase kinase 3‐dependent degradation of the Nrf2 transcription factor in a Keap1‐independent manner. Mol. Cell. Biol. 31:1121–1133. 21245377 10.1128/MCB.01204-10 PMC3067901

[26] Chowdhry, S. , Y. Zhang , M. McMahon , C. Sutherland , A. Cuadrado , and J. D. Hayes . 2013 Nrf2 is controlled by two distinct beta‐TrCP recognition motifs in its Neh6 domain, one of which can be modulated by GSK‐3 activity. Oncogene 32:3765–3781. 22964642 10.1038/onc.2012.388 PMC3522573

[27] Totoki, Y. , K. Tatsuno , K. R. Covington , H. Ueda , C. J. Creighton , M. Kato , et al. 2014 Trans‐ancestry mutational landscape of hepatocellular carcinoma genomes. Nat. Genet. 46:1267–1273. 25362482 10.1038/ng.3126

[28] Zavattari, P. , A. Perra , S. Menegon , M. A. Kowalik , A. Petrelli , M. M. Angioni , et al. 2015 Nrf2, but not *β*‐catenin, mutation represents an early event in rat hepatocarcinogenesis. Hepatology 62:851–862. 25783764 10.1002/hep.27790

[29] Nault, J. C. , J. Calderaro , L. Di Tommaso , C. Balabaud , E. S. Zafrani , P. Bioulac‐Sage , et al. 2014 Telomerase reverse transcriptase promoter mutation is an early somatic genetic alteration in the transformation of premalignant nodules in hepatocellular carcinoma on cirrhosis. Hepatology 60:1983–1992. 25123086 10.1002/hep.27372

[30] Nault, J. C. , S. Rebouissou , and J. Zucman Rossi . 2015 NRF2/KEAP1 and Wnt/beta‐catenin in the multistep process of liver carcinogenesis in humans and rats. Hepatology 62:677–679. 25846427 10.1002/hep.27828

[31] Yoo, N. J. , H. R. Kim , Y. R. Kim , C. H. An , and S. H. Lee . 2012 Somatic mutations of the KEAP1 gene in common solid cancers. Histopathology 60:943–952. 22348534 10.1111/j.1365-2559.2012.04178.x

[32] Cleary, S. P. , W. R. Jeck , X. Zhao , K. Chen , S. R. Selitsky , G. L. Savich , et al. 2013 Identification of driver genes in hepatocellular carcinoma by exome sequencing. Hepatology 58:1693–1702. 23728943 10.1002/hep.26540 PMC3830584

[33] MacLeod, A. K. , M. McMahon , S. M. Plummer , L. G. Higgins , T. M. Penning , K. Igarashi , et al. 2009 Characterization of the cancer chemopreventive NRF2‐dependent gene battery in human keratinocytes: demonstration that the KEAP1‐NRF2 pathway, and not the BACH1‐NRF2 pathway, controls cytoprotection against electrophiles as well as redox‐cycling compounds. Carcinogenesis 30:1571–1580. 19608619 10.1093/carcin/bgp176 PMC3656619

[34] Agyeman, A. S. , R. Chaerkady , P. G. Shaw , N. E. Davidson , K. Visvanathan , A. Pandey , et al. 2012 Transcriptomic and proteomic profiling of KEAP1 disrupted and sulforaphane‐treated human breast epithelial cells reveals common expression profiles. Breast Cancer Res. Treat. 132:175–187. 21597922 10.1007/s10549-011-1536-9 PMC3564494

[35] Matkowskyj, K. A. , H. Bai , J. Liao , W. Zhang , H. Li , S. Rao , et al. 2014 Aldoketoreductase family 1B10 (AKR1B10) as a biomarker to distinguish hepatocellular carcinoma from benign liver lesions. Hum. Pathol. 45:834–843. 24656094 10.1016/j.humpath.2013.12.002 PMC4030546

[36] Hayes, J. D. , and A. T. Dinkova‐Kostova . 2014 The Nrf2 regulatory network provides an interface between redox and intermediary metabolism. Trends Biochem. Sci. 39:199–218. 24647116 10.1016/j.tibs.2014.02.002

