# Supplementary figures and images for: Clinical implication of Keap1 and phosphorylated Nrf2 expression in hepatocellular carcinoma
Source: Cancer Med. 2016 Sep 20;5(10):2678–87. doi: 10.1002/cam4.788 (PMC5083719; doi:10.1002/cam4.788)

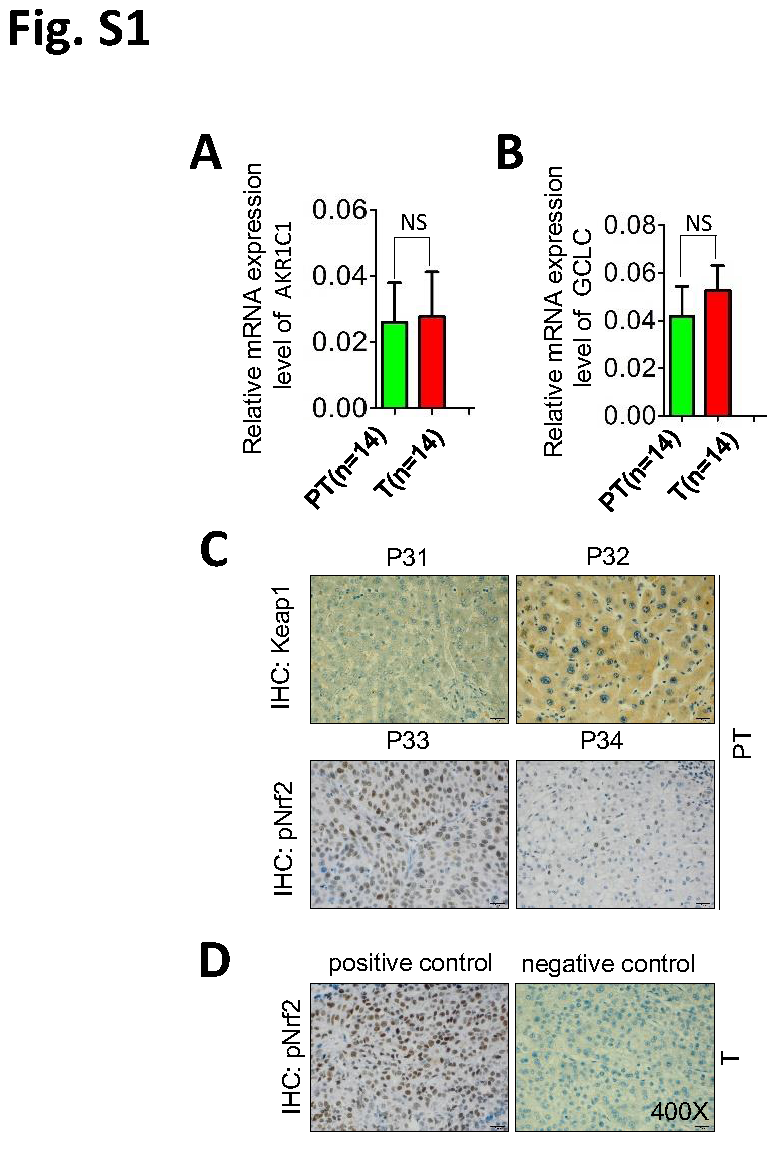

Supplement: Supplementary file 1 — Figure S1. The mRNA levels of AKR1C1(A) and GCLC(B) were assessed by qPCR in tumor(n = 14) and paratumor(n = 14). Typical staining for high and low nuclear pNrf2 or Keap1 expression in paratumor(C). The negative and positive control for the pNrf2 (Ser40) antibody (D) [file CAM4-5-2678-s001.tiff]
